# Diagnostic performance of the Xpert Carba-R assay for active surveillance of rectal carbapenemase-producing organisms in intensive care unit patients

**DOI:** 10.1186/s13756-019-0579-2

**Published:** 2019-07-29

**Authors:** Young Jin Ko, Jeeyong Kim, Ha-Nui Kim, Soo-Young Yoon, Chae Seung Lim, Chang Kyu Lee

**Affiliations:** 1 Department of Laboratory Medicine, Korea University College of Medicine, 126-1, Anam-dong 5-ga, Seongbuk-gu, Seoul, 02841 Republic of Korea; 2 Present address: Department of Laboratory Medicine, Chosun University Hospital, Gwangju, Republic of Korea

**Keywords:** Carbapenemase, Surveillance, Xpert Carba-R assay, Gram-negative bacteria, Intensive care unit, Molecular methods, Chromogenic culture

## Abstract

**Background:**

There are growing concerns regarding the spread of carbapenemase-producing organisms (CPOs) among patients in long-term care facilities (LTCFs) and hospitals in South Korea. We have established a screening protocol for the detection of CPOs in high-risk patients upon admission to intensive care units (ICUs). The diagnostic performance of the Xpert Carba-R assay was compared to that of rectal culture for CPO detection in high-risk patients upon ICU admission.

**Methods:**

A total of 408 consecutive rectal swabs were obtained from December 2016 to December 2017. CPO screening was performed using the Xpert Carba-R assay (Cepheid, Sunnyvale, CA, USA). When a carbapenemase gene was detected, additional rectal swabs were incubated overnight and inoculated on chromID CARBA medium (bioMérieux, Marcy l’Etoile, France). Bacterial carbapenemase genes, including *bla*_KPC_, *bla*_NDM_, *bla*_VIM_, *bla*_IMP-1_, and *bla*_OXA-48_, were confirmed by conventional PCR. The diagnostic performance of the Carba-R assay was ascertained based on the culture results.

**Results:**

The prevalence of CPO carriage was 7.4% according to the Carba-R assay and 3.7% according to rectal culture. The median Ct values of IMP-1 and KPC were significantly different (35.2 vs. 26.6, *P* = 0.0143). The overall sensitivity, specificity, positive predictive value (PPV) and negative predictive value (NPV) of the Carba-R assay were 100.0% (95% confidence interval [CI], 78.2–100.0), 96.7% (94.4–98.2), 53.6% (40.4–66.4) and 100.0% (99.0–100.0), respectively.

**Conclusions:**

We demonstrated the prevalence of CPO carriage in high-risk patients upon ICU admission and evaluated the diagnostic performance of the Carba-R assay. The combined use of the Xpert Carba-R assay and culture produces rapid and reliable results for the active surveillance of rectal CPO in ICU patients.

## Background

**Fig. 1:**
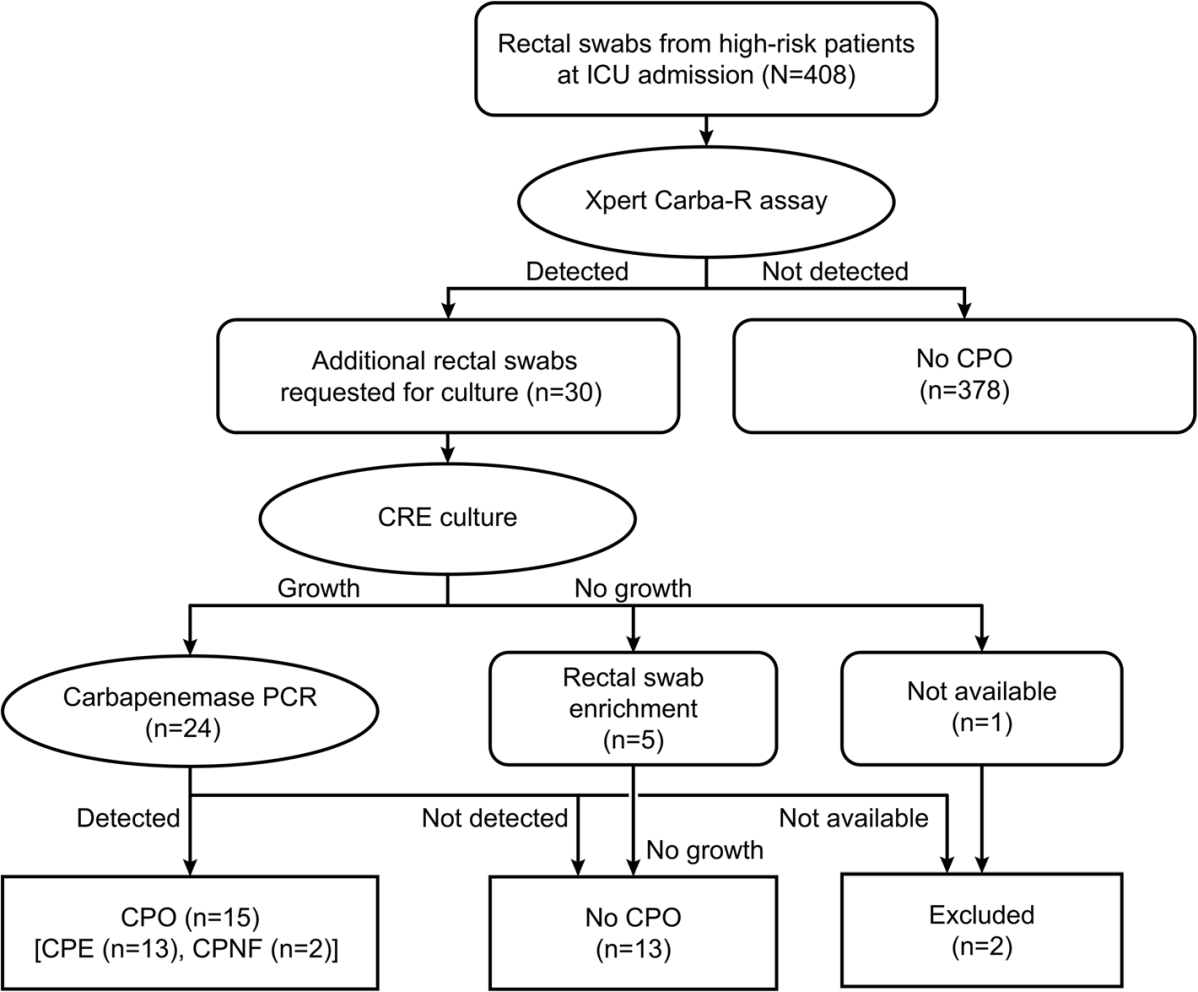
Screening protocols for carbapenemase-producing organisms in this study

The global transmission of carbapenem-resistant Enterobacteriaceae (CRE) has accelerated and become a serious public health threat worldwide [1, 2]. Carbapenemase-producing organisms (CPOs) include carbapenemase-producing Enterobacteriaceae (CPE) and carbapenemase-producing glucose nonfermenting gram-negative bacilli (CP-NF), both of which threaten human health [3]. In Europe, the prevalence of CPE is 1.3 per 10,000 hospital admissions, and the resistance rate of last-resort antibiotics is high [4]. In South Korea, high rates of carbapenem resistance among glucose nonfermenting gram-negative bacilli (CR-NF) and the increased prevalence of CRE have become serious problems in hospitalized patients [5, 6]. Since November 2010, nationwide sample surveillance has been conducted, and the number of CPE cases reported in South Korea has been dramatically increasing every year [7]. The transmission of CPE and associated outbreaks have become great concerns because of the difficulties associated with their control in both acute care hospitals and long-term care facilities (LTCFs) [6, 8]. Risk factors associated with CPE include intensive care therapy, hospitalization in the previous six months, hospital acquisition and foreign travel in the previous six months [4]. We have established a screening protocol for detecting CPOs in high-risk patients upon admission to intensive care units (ICUs) using real-time PCR and rectal culture (Fig. 1). To pre-emptively isolate CPE-colonized patients, we actively screen for CPOs using the Xpert Carba-R assay (Cepheid, Sunnyvale, CA, USA), which is a rapid and easy-to-use method for detecting five common carbapenemase genes (*bla*_KPC_, *bla*_NDM_, *bla*_VIM_, *bla*_IMP-1_, and *bla*_OXA-48_). In the present study, we aimed to elucidate the prevalence of rectal CPO carriage in high-risk patients admitted to the ICU at a tertiary hospital and to evaluate the performance of this assay for detecting rectal CPOs.

## Methods

### Study design

This study was conducted as a retrospective study. From December 2016 to December 2017, rectal swabs were requested for CPO screening from patients admitted to the ICU at Korea University Medical Center (KUMC), Guro Hospital. Clinical information on the patients was collected from electronic medical records. High-risk patients were defined as patients who had been previously hospitalized in another hospital or LTCF during the three months before admission to KUMC, Guro Hospital, or patients known as CPE carriers.

### Evaluation of the limit of detection (LoD)

Before the study period, we evaluated the LoD of the Xpert Carba-R assay and culture for carbapenem-resistant organisms (CROs). Five CPO isolates were used to evaluate the carbapenemase genes *bla*_KPC_, *bla*_NDM_, *bla*_VIM_, *bla*_IMP-1_, and *bla*_OXA-48_. Monobacterial suspensions of KPC-producing *Klebsiella pneumoniae*, IMP-1-producing *Pseudomonas aeruginosa*, NDM-producing *Enterobacter aerogenes*, VIM-producing *K. pneumoniae,* and OXA-48-producing *Escherichia coli* in sterile 0.9% saline were adjusted to a 0.5 McFarland standard. Then, sterile swabs were placed in the inocula subjected to 10-fold dilutions of 2.25 × 10^4^, 2.25 × 10^3^, 2.25 × 10^2^ and 2.25 × 10^1^ CFU/swab and vortexed with the reagent for 10 s. These inocula in 1.7 mL of reagent solution were transferred into the sample chamber in cartridges. The cartridges were loaded into a GeneXpert IV instrument (Cepheid) according to the manufacturer’s instructions. The LoD claimed by the manufacturer was verified as the lowest detected concentration of each of the five CPOs.

To determine the culture LoD, inocula of 10 CPOs were subjected to 10-fold dilutions of 1 × 10^4^, 1 × 10^3^, 1 × 10^2^, 1 × 10^1^ and 1 × 10^0^ CFU/plate and inoculated on chromID CARBA medium (chromID, bioMérieux, Marcy l’Etoile, France); visible colonies were counted after 16, 24, and 48 h of incubation at 35 °C. These inocula included five KPC-producing organisms (*K. pneumoniae*, *E. coli*, *K. oxytoca*, *Citrobacter koseri*, and *E. cloacae*), two NDM producers (*E. coli* and *C. freundii*), one IMP-1-producing *E. asburiae*, one VIM-producing *K. pneumoniae* and one OXA-48-producing *E. coli*.

### CRE culture, susceptibility testing and PCR for carbapenemase genes

When carbapenemase genes were detected with the Carba-R assay, new rectal swabs were requested for culture during the study period. New swabs were incubated overnight in thioglycolate broth and inoculated into chromID. When the organisms were recovered, identification and antimicrobial susceptibility testing were performed using the Vitek MS (bioMérieux, Manchester, UK) and Vitek 2 (bioMérieux, Hazelwood, MO, USA) instruments. Disk diffusion testing was performed with disks containing 10 μg of ertapenem, meropenem and imipenem (Oxoid, Basingstoke, UK) on Muller-Hinton agar. Clinical breakpoints and screening cutoff values for CROs were based on EUCAST breakpoint tables (http://www.eucast.org/fileadmin/src/media/PDFs/EUCAST_files/Breakpoint_tables/v_9.0_Breakpoint_Tables.pdf). When CREs were detected, a modified Hodge test and a carbapenemase inhibition test were performed according to the Clinical and Laboratory Standards Institute guidelines and diagnosis guideline for CPE [9, 10]. Additionally, in-house PCR for five carbapenemase genes and the Xpert Carba-R assay were performed to confirm the CPOs. Briefly, bacterial DNA was extracted using a Genedia Mycobacteria DNA Prep Kit (Green Cross Medical Science Corp., Chungbuk, South Korea), and modified multiplex PCR for five carbapenemase genes was performed according to a previous report [11]. After the study period, for the discrepant results between the Carba-R assay and cultures, DNA was extracted from the rectal swabs and sent to a laboratory (Macrogen, Seoul, South Korea) for bidirectional sequencing analysis of the five target genes. The sequencing primers were designed to include the target regions of the Carba-R assay in the product sequence.

### Data analysis and statistics

Differences in Ct values between the CPO-recovered and non-CPO-recovered swabs were analyzed using the Mann-Whitney test. The sensitivity, specificity, positive predictive value (PPV) and negative predictive value (NPV) were also calculated. Receiver operating characteristic (ROC) curve analysis and diagnostic performance analysis were performed using MedCalc 14.23 (MedCalc Software, Ostend, Belgium).

## Results

### Evaluation of the LoD for the Carba-R assay and CRE culture

**Fig. 2:**
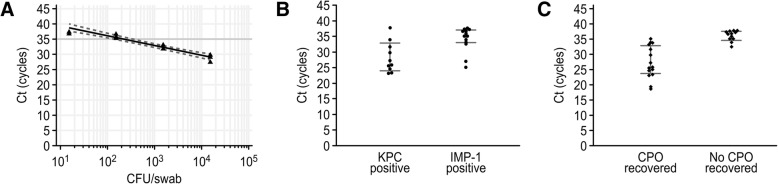
LoD of the Carba-R assay and comparison of the Ct values combined with CRE cultures. Regression analysis of the Ct values from the Carba-R assay corresponding to bacterial concentrations of 10^4^, 10^3^, 10^2^ and 10^1^ CFU/swab (**a**). The gray line indicates a Ct value of 35 cycles. Comparison of the Ct values from KPC-positive and IMP-1-positive rectal swabs (**b**). Comparison of the Ct values from rectal swabs with and without CPO recovery (**c**)

The highest Ct values measured for the five CPE isolates were 36.5, 37.7, 32.2, 36.9 and 37.0 for the detection of *bla*_KPC_, *bla*_NDM_, *bla*_VIM_, *bla*_IMP-1_ and *bla*_OXA-48_, respectively, corresponding to 225, 22.5, 2,250, 225 and 225 CFU/swab. Regression analysis generated an equation for the assumed bacterial concentration based on the assigned Ct values (Fig. 2a). According to the equation, the Carba-R assay detected Ct values less than 38.0, corresponding to a bacterial load greater than 46 CFU/swab.

The LoD of chromID for KPC-producing bacteria ranged from 1 to 10 CFU/plate, whereas the LoD for metallo-β-lactamase (MBL)-producing bacteria ranged from 10 to 100 CFU/plate. The LoD of chromID for OXA-48-producing bacteria was 1 × 10^4^ CFU/plate (Table 1).

**Table 1:** The detection limit of CRE culture stratified by carbapenemase genes

Carbapenemases	Strains	Disk diffusion (mm)			The lowest detection limit of CRE culture (CFU/plate)
		Ertapenem	Imipenem	Meropenem	
Ambler class A carbapenemase					
KPC	*C. koseri* 150	15	18	18	1
KPC	*E. cloacae* 207	7	19	15	10
KPC	*K. oxytoca* 211	18	22	23	1
KPC	*K. pneumoniae* 222	11	17	13	1
KPC	*E. coli* 223	15	17	20	1
MBL carbapenemase					
NDM	*C. freundii* 148	18	20	19	100
NDM	*E. coli* 192	6	12	10	10
VIM	*K. pneumoniae* 225	18	19	19	10
IMP-1	*E. asburiae* 44	16	21	19	100
Ambler class D carbapenemase					
OXA-48	*E. coli* 229	19	22	23	1 × 10^4^

Abbreviations: *MBL* metallo-β-lactamase

### Prevalence of rectal CPO carriage

A total of 408 rectal swabs were analyzed during the study period. The prevalence of rectal CPO carriage upon admission to the ICU was 7.4% (30 of 408) and 3.7% (15 of 406) among high-risk patients according to the Carba-R assay and culture results, respectively. IMP-1 (13, 3.2%) and KPC (10, 2.5%) were predominantly detected by the Carba-R assay, followed by NDM (4, 1.0%), VIM (0.2%), KPC with OXA-48 (0.2%) and KPC with IMP-1 (0.2%) (Table 2).

**Table 2:** Distribution of carbapenemase genes detected by the Xpert Carba-R assay and the recovered carbapenemase-producing organisms

Carbapenemase genes (No. of positive results from the Carba-R assay)	Recovered strains from additional rectal culture	PCR confirmation of the CPO strains		No. of patients	No. of CPO strains
*bla*_KPC_ (*n* = 10)	*Klebsiella pneumoniae*	KPC	CPE	5	5
	*Escherichia coli*	KPC	CPE	2	2
	*Klebsiella pneumoniae* *Escherichia coli*	KPC KPC	CPE	1	2
	No growth	No growth	No CPO	1	0
	Not available	NA	NA	1	0
*bla*_KPC_, *bla*_IMP-1_ (*n* = 1)	*Escherichia coli* *Citrobacter koseri*	KPC KPC	CPE	1	2
*bla*_KPC_, *bla*_OXA-48_ (*n* = 1)	*Klebsiella pneumoniae* *Pseudomonas aeruginosa*	KPC Negative	CPE	1	1
*bla*_NDM_ (*n* = 4)	*Escherichia coli*	KPC	CPE	1	1
	*Klebsiella pneumoniae*	NDM	CPE	1	1
	*Pseudomonas aeruginosa*	Negative	No CPO	1	0
	*Klebsiella pneumoniae*	Negative	No CPO	1	0
*bla*_VIM_ (*n* = 1)	*Klebsiella pneumoniae* *Escherichia coli*	KPC Negative	CPE	1	1
*bla*_IMP-1_ (*n* = 13)	*Pseudomonas aeruginosa*	IMP-1	CP-NF	2	2
	*Acinetobacter baumannii*	Negative	No CPO	4	0
	*Pseudomonas aeruginosa*	Negative	No CPO	2	0
	*Pseudomonas aeruginosa*	NA	NA	1	0
	No growth	No growth	No CPO	4	0

Abbreviations: *CPO* carbapenemase-producing organism, *CPE* carbapenemase-producing Enterobacteriaceae, *CP-NF* carbapenemase-producing glucose nonfermenting gram-negative bacilli, *NA* not available

Seventeen CPO strains were recovered from newly collected 29 rectal swabs. Twelve KPC-producing organisms were recovered from 11 KPC-positive rectal swabs, including 7 *K. pneumoniae* strains, four *E. coli* strains, and one *C. koseri* strain. One KPC-producing *K. pneumoniae* and one KPC-producing *E. coli* strain were recovered from KPC-negative/other gene-positive rectal swabs. Among the four NDM-positive rectal swabs, one NDM-producing *K. pneumoniae* strain was recovered. For most IMP-1-positive rectal swabs, the recovered bacteria were not CPOs, except two IMP-1-producing *P. aeruginosa* strains. VIM- or OXA-48-producing organisms were not recovered during the study period (Table 2). KPC producers (12, 3.0%) were predominantly recovered, followed by IMP-1 producers (2, 0.5%) and NDM producer (1, 0.2%).

### Diagnostic performance of the Carba-R assay

In the Carba-R assay, Ct values higher than 35.0 for rectal swabs corresponded to bacterial concentrations of less than 390 CFU/swab, and most of these swabs did not produce CRE culture growth during the study period (Fig. 2). The median Ct values of the Carba-R assay were significantly different between KPC- and IMP-1-producing organisms (26.6 vs. 35.2, *P* = 0.0143) (Fig. 2b). Additionally, the median Ct values of the assay were significantly different between rectal swabs containing CPOs and non-CPO rectal swabs (25.9 vs. 36.7, *P* = 0.0001) (Fig. 2c).

The overall sensitivity, specificity, PPV and NPV were 100.0, 96.7, 53.6, and 100.0%, respectively, when the Ct value recommended by the manufacturer (38.0 cycles) was applied as the cutoff (Table 3). Because of the differences in prevalence among carbapenemase genes, the diagnostic performance varied according to the type of carbapenemase gene detected. Due to the small number of detected organisms, the diagnostic performance of VIM and OXA-48 in terms of sensitivity and PPV could not be calculated.

**Table 3:** Diagnostic performance of the Carba-R assay stratified by carbapenemase gene

Carbapenemase gene (No. of carbapenemase genes detected by the Carba-R assay)	CPO carriage by culture (%)	No. of specimens with the following results					% (95% confidence interval)			
		Total	TP	FP	TN*	FN	Sensitivity	Specificity	PPV	NPV
KPC (*n* = 11)**†	3.0	406	10	1	393	2	83.3 (51.6–97.9)	99.8 (98.6–100.0)	90.9 (58.2–98.6)	99.5 (98.2–99.9)
NDM (*n* = 4)	0.2	406	1	3	402	0	100.0 (2.5–100.0)	99.3 (97.9–99.8)	21.3 (8.1–45.5)	100.0 (99.1–100.0)
VIM (*n* = 1)	NA	406	0	1	405	0	NA	99.8 (98.6–100.0)	NA	100.0 (99.1–100.0)
IMP-1 (*n* = 13)**†	0.5	406	2	11	393	0	100.0 (15.8–100.0)	97.3 (95.2–98.6)	15.6 (9.3–24.8)	100.0 (99.1–100.0)
OXA-48 (*n* = 1)**	NA	406	0	1	405	0	NA	99.8 (98.6–100.0)	NA	100.0 (99.1–100.0)
Total (*n* = 28)	3.7	406	15	13	378	0	100.0 (78.2–100.0)	96.7 (94.4–98.2)	53.6 (40.4–66.4)	100.0 (99.0–100.0)

*Specimens not detected by the Carba-R assay were considered as true negative results

**Two specimens were detected as KPC + IMP-1 and KPC + OXA-48

†The other two specimens were excluded because the results of PCR or CRE culture were not available.

Abbreviations: *CPO* carbapenemase-producing organism, *TP* true positive, *FP* false positive, *TN* true negative, *FN* false negative, *PPV* positive predictive value, *NPV* negative predictive value

The overall specificity and PPV were improved in the ROC curve analysis when a diagnostic cutoff value of 35.0 cycles was applied (Table 4). The areas under the ROC curve (AUCs) were not significantly different in a pairwise comparison of ROC curves when 38.0 and 35.0 cycles were used as cutoff values (0.98 vs. 0.96, *P* = 0.5405). Using a cutoff value of 35.0 cycles, the overall specificity/PPV improved from 96.7%/53.6% to 99.2%/82.4%, with minimal differences in the AUC.

**Table 4:** ROC curve analysis of the Carba-R assay stratified by carbapenemase gene and the cutoff Ct value

Carbapenemase gene	Cutoff Ct value (cycles)	% (95% confidence interval)				AUC
		Sensitivity	Specificity	PPV	NPV	
KPC	≤38.0	83.3 (51.6–97.9)	99.8 (98.6–100.0)	90.9 (58.7–99.8)	99.5 (98.2–99.9)	0.92 (0.88–0.94)*
	≤35.0	83.3 (51.6–97.9)	100.0 (99.1–100.0)	100.0 (69.2–100.0)	99.5 (98.2–99.9)	0.92 (0.89–0.94)*
IMP-1	≤38.0	100.0 (15.8–100.0)	97.5 (95.5–98.8)	16.7 (2.1–48.4)	100.0 (99.1–100.0)	0.99 (0.97–1.00)*
	≤35.0	50.0 (1.3–98.7)	99.3 (97.8–99.8)	25.0 (0.6–80.6)	99.8 (98.6–100.0)	0.75 (0.70–0.79)
Total	≤38.0	100.0 (78.2–100.0)	96.7 (94.4–98.2)	53.6 (33.9–72.5)	100.0 (99.0–100.0)	0.98 (0.97–0.99)*
	≤35.0	93.33 (68.1–99.8)	99.2 (97.8–99.8)	82.4 (56.6–96.2)	99.7 (98.6–100.0)	0.96 (0.94–0.98)*

*P < 0.0001, Abbreviations: *PPV* positive predictive value, *NPV* negative predictive value, *AUC* area under the ROC curve

## Discussion

We actively screened for rectal CPOs to support the pre-emptive isolation of ICU patients, with the results showing that the prevalence of rectal CPOs in high-risk patients using the Carba-R assay was similar to that of a previous report in South Korea [5]. The prevalence of rectal CPO carriage varied by region and was observed in 0.1% of UK hospitals in 2015 and 19.2% of Spanish hospitals in 2017 [12, 13]. The distribution of carbapenemase also exhibited regional variance. In Romania, Spain, and Turkey, OXA-48 was most frequently detected among *K. pneumoniae*, while KPC was the most detected carbapenemase in South Korea, Greece, Italy, and Portugal [4, 6].

The Ct values of the Carba-R assay in this study could predict CPO recovery in culture. The cutoff Ct value was previously reported to distinguish false-positive or insignificant results from true CPO results [14]. The cutoff value for CPO recovery in this study was 35.0 cycles, which corresponded to a bacterial concentration of 390 CFU/swab. The concentration of *bla*_IMP-1_ in most specimens was assumed to be less than 390 CFU/swab in this study. However, the sensitivity of IMP-1 decreased from 100.0 to 50.0% when using the adjusted cutoff value (35.0), which resulted from the recovery of one IMP-1 positive specimen with a Ct value of 35.1. Therefore, further studies are needed since there was an insufficient number of recovered IMP-1 producers to determine the recovery cutoff.

The type of carbapenemase gene may affect the CPO recovery of chromID CARBA medium (chromID). Our LoD values for chromID ranged from 1 to 10 CFU/plate for KPC producers, 10 to 100 CFU/plate for MBL carbapenemase producers, and the LoD for OXA-48 producers was 1 × 10^4^ CFU/plate. These results are consistent with a previous report in which the LoD for KPC-producing bacteria was 1 × 10^1^ CFU/plate, with values of 1 × 10^1^ to 1 × 10^6^ CFU/plate for NDM- and VIM-producing bacteria and 1 × 10^5^ CFU/plate for OXA-48-producing bacteria [15]. ChromID is known to be insufficient for detecting OXA-48-producing bacteria because of its low analytic sensitivity [14]. However, we did not use ChromID OXA-48 medium during the study period due to the low incidence of OXA-48-producing strains in our hospital.

We frequently detected the *bla*_IMP-1_ gene in this study, with a detection rate of 3.2%; however, most of the isolated bacteria did not contain this gene. Only two strains of IMP-1-producing *P. aeruginosa* were recovered from 13 IMP-1-positive rectal swabs. *P. aeruginosa* is usually present at lower concentrations than other gram-negative bacteria in the perianal region [16]. Therefore, a low concentration of *P. aeruginosa* can be considered a major cause of inconsistency with culture results. The Carba-R assay can detect the IMP-1, IMP-3, IMP-6, IMP-10, IMP-25, and IMP-30 subgroups without distinction. Notably, IMP-6-producing *P. aeruginosa* has become a predominant clone, and IMP-10-producing *P. aeruginosa* has recently emerged in South Korea [17, 18]. In addition, most of the carbapenemase-producing *P. aeruginosa* isolates were previously observed to exhibit extensive drug resistance and showed susceptibility only to colistin in 2015 [18].

We found four NDM-positive cases, from which only one NDM-producing bacterial strain was cultured. To address this discrepancy, three NDM- and three IMP-1-positive/culture-negative specimens were collected, and direct PCR sequencing of the five carbapenemase genes was performed. We detected *bla*_NDM_ sequences in one NDM-positive swab, from which meropenem-susceptible *K. pneumoniae* was recovered. This isolate showed weak positivity in the Hodge test, resistance to ertapenem, and susceptibility to imipenem. We also found a *bla*_IMP-1_ sequence in one IMP-1-positive swab from which bacteria were not recovered. This sequence showed 99.1% homology with *bla*_IMP-1_, *bla*_IMP-14_, *bla*_IMP-48_ and *bla*_IMP-54_. No other carbapenemase gene sequences were found in the other specimens. In summary, the low concentration of CPOs in rectal swabs, the false-positive results obtained for NDM and IMP-1 using the Carba-R assay and the weak hydrolysis of MBL carbapenemase were the primary causes of discordance between the Carba-R assay and culture results in this study.

Most studies have validated and evaluated the Carba-R assay using contrived specimens of *bla*_IMP-1_ producers or pure colonies [19–22]. All five types of carbapenemase genes, including *bla*_IMP-1_, were detected with a low prevalence in this study. In addition, the clinical performance of the Carba-R assay using rectal swabs for active surveillance was reliable and concordant with that of a previous report, except for the PPV of the *bla*_IMP-1_ and *bla*_NDM_ genes [19, 20]. The combined use of the Carba-R assay and culture was found to be a sensitive and specific screening method for CPOs [22]. This combination is advantageous because the molecular method detects only the target gene and allows false-positive results, whereas rectal culture can identify which bacteria are carbapenem-resistant but may be less sensitive because of the abundance of other enteric bacteria [23]. The Carba-R assay enables the detection of most carbapenemase genes, including the recently emerging *bla*_OXA-181_ and *bla*_OXA-232_ genes; however, *bla*_GES_ cannot be detected by the assay [24]. Thus, regional epidemiology should be considered when choosing a screening method for CPO detection [22].

To prevent CPO transmission, all ICU patients were pre-emptively isolated on the day of admission and subsequently released from quarantine according to the Carba-R assay result, which was reported daily. For the patients who tested positive by the Carba-R assay, the quarantine was released after three consecutive negative CRE culture results. This protocol shortened the period of unnecessary patient isolation and reduced medical staff fatigue.

This study has several limitations. We compared the results of the Carba-R assay and CRE cultures for carbapenemase gene-positive specimens. The Carba-R assay was an imperfect reference method which had the possibility of false negativity, especially for a *bla*_KPC_ gene. After the study period, we compared the Carba-R assay and CRE culture results for 100 PCR-negative specimens. CRE was not cultured for any of the PCR-negative specimens. Among the specimens, five strains of carbapenem-susceptible *P. aeruginosa* and one strain of carbapenem-resistant *A. baumannii* complex were recovered. No carbapenemase gene was detected in the strain. The Carba-R assay of prospectively collected rectal swabs from the previous study also did not have a false-negative result [23].

The Carba-R assay did not detect *bla*_GES_, and we could not exclude the presence of *bla*_GES_ in this study. However, regarding the local epidemiology near the hospital, *bla*_GES_ and other carbapenemase genes have not been reported [7]. The low prevalence of *bla*_VIM_ and *bla*_OXA-48_ limited the calculation of the sensitivity and PPV in this study. Further large-scale studies directly comparing culture combined with direct PCR-sequencing and the Carba-R assay for CPO detection will elucidate these limitations.

## Conclusions

We demonstrated the prevalence of rectal CPO carriage in high-risk patients upon admission to the ICU in a tertiary hospital and evaluated the diagnostic performance of the Xpert Carba-R assay. The combined use of the Xpert Carba-R assay and culture produces rapid and reliable results for active surveillance of rectal CPO in ICU patients. The use of cutoff Ct values to improve the specificity of the Xpert Carba-R assay was applicable for decision-making associated with patient isolation.

## Data Availability

Not applicable.

## Abbreviations

AST: Antimicrobial susceptibility test
AUC: Area under the ROC curve
CFU: Colony forming unit
CPE: Carbapenemase-producing Enterobacteriaceae
CP-NF: Carbapenemase-producing glucose nonfermenting gram-negative bacilli
CPO: Carbapenemase-producing organism
CR-NF: Carbapenem-resistant glucose nonfermenting gram-negative bacilli
CRO: Carbapenem-resistant organism
Ct: threshold cycle
EUCAST: The European Committee on Antimicrobial Susceptibility Testing
FN: False negative
FP: False positive
ICU: Intensive care unit
IMP: Imipenemase
KPC: *Klebsiella pneumoniae* carbapenemase
LTCF: Long-term care facility
MBL: Metallo-β-lactamase
NA: Not available
NDM: New Delhi metallo-β-lactamase
NPV: Negative predictive value
OXA: Oxacillinase
PPV: Positive predictive value
ROC: Receiver operating characteristic
TN: True negative
TP: True positive
VIM: Verona integron-encoded metallo-β-lactamase
